# Results after Repair of Functional Tricuspid Regurgitation with a Three-Dimensional Annuloplasty Ring

**DOI:** 10.3390/jcm10215080

**Published:** 2021-10-29

**Authors:** Elda Dzilic, Thomas Guenther, Amel Bouziani, Bernhard Voss, Stephanie Voss, Keti Vitanova, Markus Krane, Ruediger Lange

**Affiliations:** 1 Department of Cardiovascular Surgery, German Heart Centre Munich, Technische Universität München, 80636 Munich, Germany; guenther@dhm.mhn.de (T.G.); bouziani-amel@hotmail.fr (A.B.); voss@dhm.mhn.de (B.V.); grabert@dhm.mhn.de (S.V.); vitanova@dhm.mhn.de (K.V.); lange@dhm.mhn.de (R.L.); 2 Insure (Institute for Translational Cardiac Surgery), Department of Cardiovascular Surgery, German Heart Centre Munich, Technische Universität München, 80636 Munich, Germany; 3 Division of Cardiac Surgery, Department of Surgery, Yale University School of Medicine, New Haven, CT 06520, USA; markuskrane@gmx.de; 4 German Heart Center Munich—DZHK Partner Site Munich Heart Alliance, 80636 Munich, Germany

**Keywords:** tricuspid valve repair, annuloplasty ring, functional tricuspid regurgitation

## Abstract

Background: Tricuspid valve (TV) repair is the recommended treatment for severe functional tricuspid regurgitation (fTR) in patients undergoing left-sided surgery. For this purpose, a wide range of annuloplasty devices differing in form and flexibility are available. This study reports the results using a three-dimensional annuloplasty ring (Medtronic, Contour 3D Ring) for TV repair and analysis of risk factors. Methods: A cohort of 468 patients who underwent TV repair (TVr) with a concomitant cardiac procedure from December 2010 to January 2017 was retrospectively analyzed. Results: At follow-up, 96.1% of patients had no/trivial or mild TR. The 30-day mortality was 4.7%; it significantly differed between electively performed operations (2.7%) and urgent/emergent operations (11.7%). Risk factors for recurrent moderate and severe TR were LVEF < 50%, TAPSE < 16 mm, and moderate mitral valve (MV) regurgitation at follow-up. Preoperatively reduced renal function lead to a higher 30-day and overall mortality. Reoperation of the TV was required in six patients (1.6%). Risk factors for TV related reoperations were preoperative TV annulus over 50 mm and an implanted permanent pacemaker. Conclusions: TVr with the Contour 3D annuloplasty ring shows low TR recurrence and reoperation rates. Risk-factor analysis for the recurrence of TR revealed the importance of left- and right-ventricular function.

## 1. Introduction

The surgical treatment of functional tricuspid valve (TV) regurgitation has moved into the spotlight since numerous studies have shown that untreated tricuspid regurgitation (TR) during left-sided valve surgery leads to progressive TR and increased mortality [1,2,3,4]. Hence, the previously prevailing idea that functional TR (fTR) may improve without intervention after treating the left-sided valve disease has shifted [5]. Hence, the 2021 ESC/EACTS guidelines still recommend TV repair (TVr) for patients undergoing left-sided valvular surgery in the presence of moderate/severe tricuspid insufficiency (recommendation class I, level of evidence B) or isolated annular dilation (>40 mm), even in the absence of severe TR (recommendation class IIa, level of evidence B) [6]. Moreover, surgery should be considered in patients with fTR who are symptomatic or have right ventricular dilatation, in the absence of severe right or left ventricular dysfunction and severe pulmonary vascular disease/hypertension (recommendation class IIa, level of evidence B) [6].

The surgical procedures for TR parallel the techniques for treating mitral insufficiency [7], in particular the use of an annuloplasty ring [8,9,10]. Currently, several annuloplasty devices with different features are available: flexible, semirigid, or rigid combined with planar and 3D geometry. The choice of device is mainly based on the surgeon’s preference.

Characteristic of fTR consists of annular dilatation with the loss of the normal 3D profile resulting in a more circular and flattened annulus leading to impaired leaflet coaptation [11]. In order to restore the natural geometrical shape, 3D annuloplasty rings have been developed. Experimental investigation in mitral valve (MV) repair using similar 3D devices shows that the coaptation surface may be improved by contoured devices [12]. Additionally, 3D annuloplasty rings result in an increased leaflet curvature reducing transmitted tension to the MV [12]. Potentially, this may increase the durability of the reconstructive procedure. First results using 3D annuloplasty devices have shown promising results [13,14,15].

In August 2010, the Contour 3D ring (Medtronic, Minneapolis, MN, USA) was introduced. It is a rigid, incomplete annuloplasty ring that resembles the 3D shape of the physiologic TV annulus in mid-systole. In this study, we present our results using the Contour 3D annuloplasty ring in a large patient cohort and analyze the risk factors for TR recurrence.

## 2. Materials and Methods

This study presents a retrospective analysis of all consecutive patients, who underwent TVr for fTR with the Contour 3D annuloplasty ring with concomitant cardiac procedures at the German Heart Center Munich between December 2010 and January 2017. Patients with isolated TVr or endocarditis were excluded from the study. Patients’ data were identified from our internal clinical database. This investigation was approved by the Ethics Committee of the Faculty of Medicine of the Technische Universität München (no. 180/14).

Endpoints. The primary endpoint of this study was TR recurrence after TVr. Secondary endpoints were survival rates and the rate of reoperation.

Operative Technique. All operations were performed on cardiopulmonary bypass under systemic hypothermia (26–32 °C). Myocardial protection was achieved using cold (4 °C) crystalloid antegrade cardioplegia (Bretschneider solution) or warm blood cardioplegia. For proper sizing of the Contour 3D ring, both the distance between the commissures of the septal leaflet and the area of the anterior leaflet were measured by using the ring sizer provided by the manufacturer. The ring is marked at three points with colored sutures, which correspond to the commissures of the TV. Intra-annular Ethibond 3.0 sutures (Ethicon, Norderstedt, Germany) were used for ring fixation. An emergent operation was defined as being required within 24 h after admission. Operations required within the same hospital stay due to severe clinical presentation of the patient were defined as urgent. The atrial lesions for the Cox maze IV procedure were performed using the Medtronic Cardioblade Cryo-Flex Device as previously described [16]. All patients were discharged on a regimen of phenprocoumon (Coumadin) for the first 3 months postoperatively. Beyond this time, anticoagulant therapy was continued only in patients with atrial fibrillation or other specific indications.

Follow-up. Complete follow-up (FU) was achieved in 95% of patients, yielding a cumulative total of 1217 patient-years and a mean FU of 2.52 ± 2.0 years. Clinical symptoms, hemodynamic data, and functional outcome were obtained from medical records, patient follow-up visits, telephone interviews, and communications from the referring physicians. FU data included current symptoms, echocardiographic results, and occurrence of late cardiac events. Echocardiographic examinations performed in our institution were done by a team of three investigators. Echocardiographic FU was obtained from in-hospital data or from cardiologists in private practice. Echocardiographic FU was achieved in 68% of patients. All in-hospital evaluations were carried out according to standard techniques recommended by the American Society of Echocardiography. TR was graded as none/trivial (0+), mild (1+), moderate (2+), or severe (3+).

Statistical Analysis. Statistical analysis was performed with the Statistical Package for Social Science (SPSS Inc., Chicago, IL, USA, version 25) for Windows and R statistical software language (R Foundation for Statistical Computing, Vienna, Austria, version 3.6.1). Comparison between groups was performed using either a Fisher’s exact test for binominal variables or t-test for normally distributed variables. Differences in the end points were evaluated using the log-rank Mantel Cox test and the cumulative incidence analysis following the Gray-test including hazard ratio (HR) with 95% confidence intervals (CIs). Risk factor analysis was performed using logistic regression model. A probability value less than 0.05 was considered to be statistically significant. All data are represented as mean ± standard deviation or as median, for actuarial estimates the standard error is reported instead.

## 3. Results

### 3.1. Preoperative Data

A total of 468 consecutive patients ≥18 years (266 men, 56.8% and 202 women, 43.2%) with an average age of 69.4 ± 9.7 (30 to 87) years were included. The study population included 275 patients (58.8%) older than 70 years and 47 (10%) older than 80 years.

Upon admission, 121 patients (25.9%) were in New York Heart Association (NYHA) functional class II, 208 (44.4%) in NYHA class III, and 105 (22.4%) in NYHA class IV. Concomitant coronary artery disease was present in 172 patients (36.8%), 37 patients (7.9%) had a history of myocardial infarction, and 41 (8.7%) had undergone a previous percutaneous coronary intervention. Preoperative echocardiographic data are summarized in Table 1. Additionally, 331 patients (78%) presented with moderate/severe MR, 41 patients (10%) with moderate/severe aortic valve regurgitation, and 60 patients (19%) with moderate/severe aortic valve stenosis.

Atrial fibrillation was present in 288 patients (61.6%) and a permanent pacemaker and/or ICD had been previously implanted in 34 (7.3%). Previous cardiac operations had been performed in 81 patients (17.3%). There was a mild to moderate reduction in the glomerular filtration rate (GFR 30–60 mL/min) in 37% of patients and severe reduction of GFR (GFR < 30 mL/min) in 4.7% of patients. Clinical and demographic data are summarized in Table 2.

### 3.2. Operative Data

An emergent or urgent operation was performed in 103 patients (22%). The majority of the patients (82.7%) underwent a concomitant MV procedure. Additionally, 22.6% of patients received a concomitant aortic valve (AV), 26.5% had a CABG procedure, and 22% of the patients received a simultaneous modified MAZE procedure. Approximately one-third (34.4%) of patients underwent more than one concomitant procedure (either MV, AV, Aorta and/or CABG) and 4.7% of patients had more than two additional procedures. Twenty-six (5.6%) were operated through a right anterolateral minithoracotomy. Detailed operative data are given in Table 3.

### 3.3. Postoperative Course

Due to hemodynamic instability, fourteen patients (3.0%) required intra-aortic balloon pump assistance and eight patients (1.7%) required ECMO assistance. The incidence of acute renal failure requiring hemodialysis was 14.7%. Twenty-one patients (4.5%) suffered from an ischemic stroke. Forty patients (8.5%) displayed a third-degree AV-block, requiring permanent pacemaker implantation (PPI). Patients undergoing modified MAZE procedure had a significantly higher rate of PPI (23.3%; *p* = 0.001). The average length of postoperative ICU stay was 9.1 ± 16.0 days (median 4). Patients were discharged after a median hospital stay of 14 days.

### 3.4. Survival

**Figure 1:**
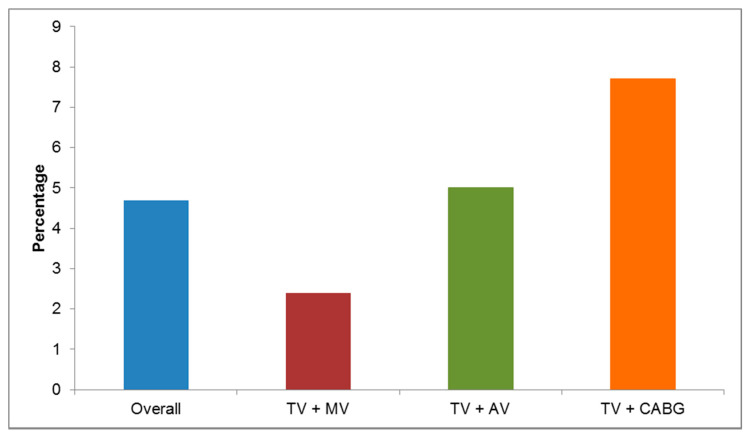
Thirty-day mortality for combined procedures. TV = tricuspid valve; MV = mitral valve; AV = aortic valve; CABG = coronary artery bypass grafting.

Surgical mortality was 0.2% (*n* = 1). This patient underwent MV replacement, TVr and CABG as urgent procedure. The cause of death was uncontrollable bleeding. The 30-day mortality was 4.7% (*n* = 22); 2.7% for electively performed operations and 11.7% for urgent/emergent operations (*p* = 0.001). The 30-day mortality varied depending on the concomitant procedures performed (Figure 1). Patients with a reduced renal function preoperatively (GFR <60 mL/min) showed a significantly higher 30-day mortality of 8.2% (*p* = 0.002).

**Figure 2:**
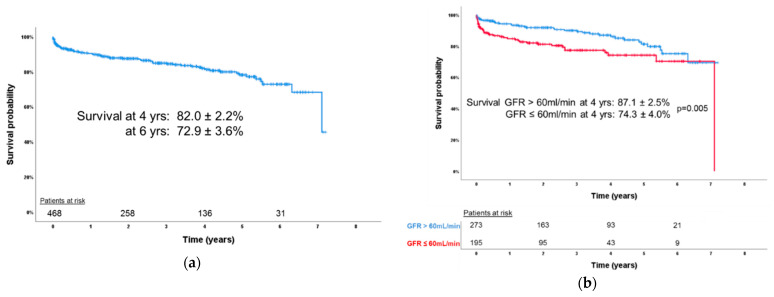
(**a**) Kaplan–Meier estimate of survival for patients after tricuspid valve repair; (**b**) Kaplan–Meier estimate of survival for patients with and without renal insufficiency.

Actuarial survival was 82.0 ± 2.2% and 72.9 ± 3.6% at 4 years and 6 years, respectively (Figure 2a). Patients with a GFR <60 mL/min showed a significantly lower actuarial survival probability at 4 years (74.3 ± 4.0% vs. 87.1 ± 2.5%, *p* = 0.005) (Figure 2b).

### 3.5. Reoperation

Twenty-six patients (5.6%) underwent cardiac reoperation for any reason. The 30-day mortality rate for these patients was 7.7%.

Six patients (1.6%) required reoperation of the TV. Table 4 shows the intraoperative findings of the TV. Half of these patients underwent TV re-repair.

**Figure 3:**
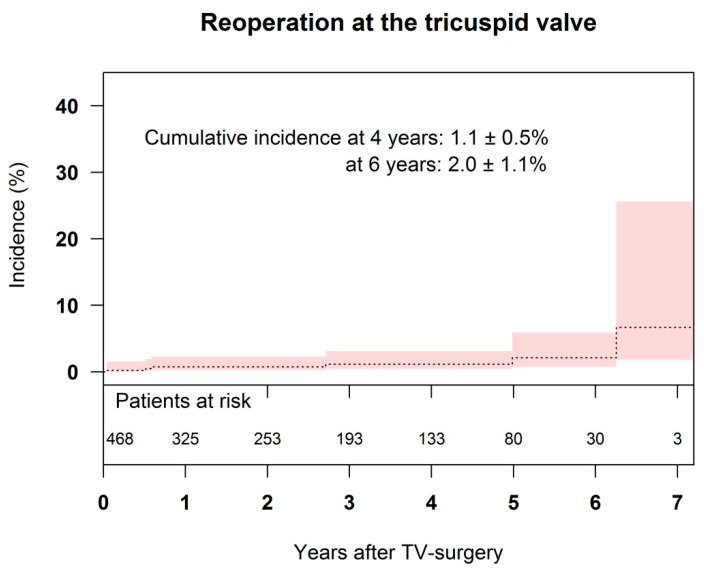
Cumulative incidence of tricuspid valve reoperation after tricuspid valve repair. TV = tricuspid valve.

Cumulative incidence for TV reoperation was 1.1 ± 0.5% after 4 years and 2.0 ± 1.1% after 6 years (Figure 3). Risk factors for TV related reoperation were preoperative TV annulus over 50 mm (*p* = 0.033) and implanted permanent pacemaker (*p* = 0.011).

### 3.6. Recurrent Tricuspid Regurgitaton

**Figure 4:**
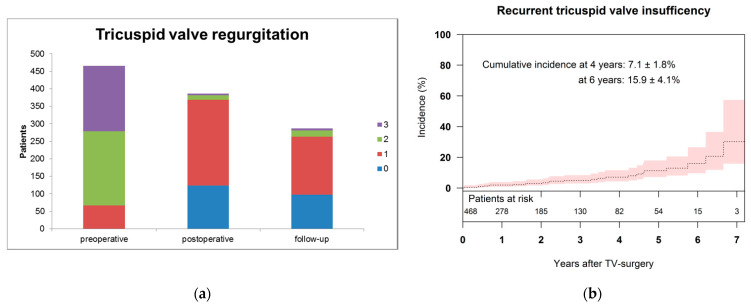
(**a**) Tricuspid valve regurgitation graded no/trivial (0), mild (1), moderate (2), and severe (3) preoperatively, postoperatively at discharge, and at latest follow-up. (**b**) Cumulative incidence of recurrent tricuspid regurgitation ≥2 after tricuspid valve repair.

Echocardiographic FU revealed that 96.1% of the patients showed no/trivial to mild TR at the latest follow-up (Figure 4a). Cumulative incidence for moderate and severe TR was 7.1 ± 1.8% and 15.9 ± 4.1% at 4 years and 6 years, respectively (Figure 4b).

Risk factors for recurrence of moderate and severe TR were LVEF < 50% (*p* = 0.005), TAPSE < 16 mm (*p* = 0.014), and moderate to severe MR at follow-up (*p* = 0.001).

## 4. Discussion

The TV has a saddle-shaped elliptical annulus, which displays dynamical changes in size and shape during the cardiac cycle. In the context of fTR, the annulus dilates mainly in the anterolateral direction of the right ventricular (RV) free wall resulting in a more circular, flattened annulus. Thus, dynamical changes during the cardiac cycle are also diminished or abolished.

The approach for TVr is based on the concept of annular remodeling, first systematically introduced by Alain Carpentier for MV disease in the 1970s and later adapted for the TV [17]. His “functional approach” of valve repair is mainly based on the aim to restore normal valve function rather than normal valve anatomy [18]. The implantation of an annuloplasty ring aimed to reduce the valvular annulus and thereby improve leaflet coaptation. Moreover, further annular dilatation is prevented.

Based on these goals, the first annuloplasty ring developed was plane and rigid, therefore disregarding the anatomical valvular shape and surrounding structures [19]. Soon thereafter, several adjustments were made to the first generation of annuloplasty rings taking valve anatomy into consideration [13,15,20,21]. Nowadays, numerous annuloplasty devices are available, differing in form and flexibility. The question of which device is preferable for sustained functional results and for RV function remains to be determined.

Nishi et al. showed that the implantation of 3D annuloplasty rings, in contrast to plane devices, leads to preserved tricuspid annular dimensions measured by annular area and the anteroposterior as well as septolateral diameters throughout the cardiac cycle [14].

These results were confirmed by an experimental sheep model [22]. After induction of right heart failure, the animals underwent a surgical procedure with the implantation of either a flexible band (Duran AnCore), a rigid 3D ring (Contour 3D), or a semi-rigid device (Tri-Ad Adams). The embedding of several sonomicrometry crystals during surgery allowed the evaluation of tricuspid annular geometry. The annular height was maintained only after implantation of the Contour 3D ring, indicating the preserved 3D geometry of the tricuspid annulus.

Experimental and clinical studies on the MV have shown that the 3D saddle-shape is important in order to reduce leaflet stress [23], restore leaflet curvature [12], increase leaflet coaptation [24], and thereby potentially improve repair durability. Corresponding investigations on the TV do not yet exist.

The design of the Contour 3D annuloplasty ring is based on CT, echo, and MRT measurements of the tricuspid annulus of healthy individuals. The shape of the prosthetic ring depicts the annulus at the time of maximal leaflet coaptation at mid-systole. The ring consists of a titanium core covered with silicon and polyester. The septolateral diameter of the device is 27% smaller compared to the physiological annular dimensions. Our initial experience with the Contour 3D annuloplasty ring was published in 2014 and showed very promising results [13].

This present retrospective analysis evaluated the mid-term results after TVr using the rigid, three-dimensional Contour 3D annuloplasty ring. In our study cohort, 96.1% of the patients showed no/trivial to mild TR at the latest follow-up (Figure 4A). Cumulative incidence for moderate and severe TR was 7.1 ± 1.8% and 15.9 ± 4.1% at 4 years and 6 years, respectively (Figure 4B). This data is in line with other studies using 3D devices, which show freedom from moderate and severe TR of about 88 to 90% after 3 to 4 years [20,21].

Risk factors for recurrence of moderate and severe TR were LVEF < 50% (*p* = 0.005), TAPSE < 16 mm (*p* = 0.014), and moderate to severe MR at follow-up (*p* = 0.001). These risk factors are solely associated with the severity of tricuspid/mitral valve disease and heart function.

In a meta-analysis of 2488 patients, Kara et al. showed that untreated mild to moderate TR at the time of mitral valve (MV) surgery evolved to a 22.6% rate of progression to moderate/severe TR during follow-up [2]. On the other hand, Chikwe et al. presented that TVr concomitant to MV repair leads to freedom of late moderate/severe TR in 97 ± 2% of cases and is associated with the recovery of right ventricular function and decrease of pulmonary artery pressure [4].

Freedom from reoperation in other studies range from 92% to 98% after 5 years [10,25,26], and are consistent with our results with a cumulative incidence for TV reoperation of 1.1 ± 0.5% after 4 years and 2.0 ± 1.1% after 6 years (Figure 3).

Risk factors for TV related reoperation were preoperative TV annulus over 50 mm (*p* = 0.033) and implanted permanent pacemaker (*p* = 0.011). Höke et al. showed that 38% of the patients developed new onset severe TR after the implantation of pacemaker or ICD leads [27]. The use of epicardial pacemakers leads or new leadless devices, such as the Medtronic Micra [9] could avoid this complication. Although, Beurskens et al. were able to show that implantation of leadless devices did not prevent the intensification of TR compared to conventional pacemakers after a follow-up of 12 months [28]. However, this study was conducted in a cohort of 53 patients without TV annuloplasty, thus leaving the question unanswered whether TV annuloplasty might prevent worsening of TR. The 30-day mortality in our study was 4.7%, which is in line with other studies (3.2–4.6%) [29]. 30-day mortality was 2.7% for electively performed operations and 11.7% for urgent/ emergent operations. Figure 1 depicts the 30-day mortality depending on the concomitant procedure. TVr combined with MV procedures show 30-day mortality of 2.4%. The mortality rate increases up to 7.7% when looking at TVr and CABG. Over a third of the patients (34.4%) had more than two concomitant procedures. A well-established risk factor for 30-day mortality and long-term survival is renal failure; this trend was also seen in this study. The 30-day mortality significantly increased in patients with a preoperatively impaired GFR (less than 60 mL/min) to 8.2%. In this study, 37% of patients showed a mild to moderate (GFR 30–60 mL/min) and 4.7% a severe reduction in glomerular filtration rate (GFR <30 mL/min) preoperatively, which is consistent with previous data from our institution [13]. Renal replacement therapy was needed in 7.9% of our patients, with a prolonged need for ventilation and longer hospital stay.

The decrease in mortality in patients with severe fTR undergoing left-sided surgery is well documented [30,31], but the less common case of fTR due to coronary artery disease lacks clear recommendations due to missing data. However, a recent single center study reported their data in regard to fTR in patients undergoing isolated CABG [32]: Only 26 of 81 patients with severe TR underwent TV surgery simultaneously to CABG. Patients with severe TR undergoing TVr at the time of CABG had decreased early morbidity and mortality compared to patients without TVr, although nonsignificant.

## 5. Conclusions

The implantation of the rigid, three-dimensional Contour 3D annuloplasty ring for TVr results in good outcomes in regard to the recurrence of TR, which are comparable to other 3D and plane annuloplasty devices. Risk-factor analysis for recurrent TR revealed the importance of ventricular function highlighting the significance of early referral to surgery prior to impairment of left and right ventricular function. However, this is challenging as TVr in this patients cohort is not the leading indication for surgical intervention. The concomitant symptoms of TR can be well alleviated through medical therapy, thereby potentially allowing functional and structural changes like RV dilatation, RV-EF impairment, or TA dilatation. Whether an earlier referral is truly beneficial in this patients cohort needs to be determined.

## 6. Limitations

Limitations of this study are consistent with those inherent to retrospective analysis in a single center study design. Additionally, the echocardiographic data at follow-up is only complete in 68% of patients and was obtained from the different cardiologists, thus limiting a standardized examination.

## Figures and Tables

**Table 1:** Preoperative echocardiographic data.

Preoperative Eechocardiographic Data	
LVEF (%)	52.5 ± 12.4 (55 (45–60))
TAPSE	20.5 ± 6.0 (20 (16–24))
TV regurgitation	
None	0 (0%)
Mild	67 (14.3%)
Moderate	211 (45.1%)
Severe	188 (40.1%)
Tricuspid maximal annular diameter:	
mm; transthoracic	37.7 ± 6.7 (37 (33–42))
mm; transoesophageal	45.4 ± 5.2 (45 (42–50)

LVEF = left-ventricular ejection fraction; TAPSE = tricuspid annular plane systolic excursion; TV = tricuspid valve. Data is presented as mean ± SD; (median (IQR)) or as *n* (%).

**Table 2:** Baseline characteristics.

Baseline Characteristics	
Patients	*n* = 468
Age (years)	69.4 ± 9.7 (71.8 (64.9–75.8))
Female	202 (43.2%)
CVRF	
Diabetes mellitus	58 (12.3%)
Arterial hypertension	370 (79.1%)
Smokers	164 (35.0%)
Dyslipidaemia	230 (49.7%)
Overweight (BMI ≥ 25 kg/m^2^)	263 (56.2%)
Atrial fibrillation	288 (61.6%)
Pulmonary hypertension	315 (67.3%)
sPAP (mmHg)	47.9 ± 16.3 (45.0 (35.0–58.0))
Serum creatinine (mg/dL)	1.2 ± 0.5 (1.0 (0.9–1.3))
GFR (mL/min)	63.8 ± 20.5 (64.4 (49.8–77.0))
Serum bilirubine mg/dL	1.0 ± 0.8 (0.8 (0.5–1.1))
INR	1.3 ± 0.5 (1.1 (1.0–1.4))
Anticoagulant therapy	262 (56.0%)
Simplified MELD Score	8.5 ± 2.7 (7.3 (6.4–9.7))
Previous cardiac surgery	81 (17.3%)
Mitral valve surgery	52 (64.2%)
Aortic valve surgery	25 (30.9%)
CABG	8 (9.9%)
Permanent pacemaker	34 (7.3%)

CVRF = cardiovascular risk factor; BMI = body mass index; sPAP = systolic pulmonary arterial pressure, measured by echocardiography; GFR = glomerular filtration rate; INR = international normalized ratio; MELD = model end stage liver disease; CABG = coronary artery bypass grafting. Data is presented as mean ± SD; (median (IQR)) or as *n* (%).

**Table 3:** Operative data.

Operative Data	
Non-elective surgery	103 (22.0%)
Right anterolateral minithoracotomy	26 (5.6%)
Cardiopulmonary bypass time, mins	148.6 ± 52.5
Cross-clamp time, mins	94.5 ± 36.4
Size implanted ring	
26	1 (0.2%)
28	18 (3.8%)
30	89 (19.0%)
32	198 (42.3%)
34	123 (26.3%)
36	39 (8.3%)

**Table 4:** Reoperations.

Reoperations		
Intraoperative Finding	Time (d)	Procedure
Gap between anterior and septal leaflet	15	TV repair
Restrictive motion of the septal leaflet	134	TV replacement
Endocarditis	189	TV replacement
Prolapse of the anterior leaflet	213	TV repair
Restrictive motion of the septal/anterior leaflet	990	TV replacement
Gap between anterior and posterior leaflet	2284	TV repair

TV = tricuspid valve.

## Data Availability

The data presented in this study are available on reasonable request from the corresponding author.
